# Germinal centre and marginal zone B cells expand quickly in a second *Plasmodium chabaudi* malaria infection producing mature plasma cells

**DOI:** 10.1111/j.1365-3024.2008.01066.x

**Published:** 2009-01

**Authors:** R STEPHENS, F M NDUNGU, J LANGHORNE

**Affiliations:** National Institute for Medical Research, Division of Parasitology, The Ridgeway, Mill HillLondon NW7 1AA, UK

**Keywords:** B lymphocyte, malaria, immunological memory, mouse

## Abstract

*Antibodies and B cells are critical in the protective immune response to the blood stage of the malaria parasite,* Plasmodium chabaudi. *However, little is known about the development of memory B cells and their differentiation into plasma cells during infection or after re-infection. Here we have shown that B cells with phenotypic characteristics of memory cells (CD19^+^IgD^−^ CD38^+^, IgG1^+^) are generated in a primary*Plasmodium chabaudi chabaudi *infection of mice. In addition, we observed that germinal centre cells (CD19^+^, GL7^+^, MHCII^hi^) and Marginal Zone B cells (CD19^+^CD23^−^IgD^−^) show faster expansion on re-infection than in the primary, though other subsets do not. Interestingly, though both IgM^−^ and IgM^+^ memory cells are produced, IgM^+^ memory cells do not expand on second infection. The second infection quickly produced mature bone marrow plasma cells (intracellular Ig^hi^, CD138^hi^, CD9^+^, B220^−^), compared to primary infection; which generates a very large population of immature splenic plasma cells (B220+). This analysis suggests that a memory B cell population is generated after a single infection of malaria, which on re-infection responds quickly producing germinal centres and generating long-lived plasma cells making the second encounter with parasite more efficient.*

## INTRODUCTION

Immunity to malaria is slow to develop, and although prevalence and levels of parasitaemia decrease with exposure, susceptibility to re-infection remains for years even after repeated exposure (1,2). This may be due to extensive antigenic variation of the parasite (3) and strain-specific immunity. However, susceptibility to repeat infections and short-lived protection from pathology may also result from inherent or malaria-specific limitations of immunological memory and protective immunity (1). Antibodies are considered to be a major effector mechanism eliminating blood-stage malaria parasites in humans (4,5), and in experimental models (6,7), and it is therefore possible that impairment of protective immunity is due to defects in the B cell response. Most of our information to date on the B cell response in both human and experimental infections has come from analysis of antimalaria antibodies in sera. While there are many studies documenting the levels, isotype and duration of the antibody responses (reviewed in Ref. (8)), there are very few cellular studies investigating the nature and development of the antibody-producing cells; B cells and plasma cells. Those studies, describing large short-lived polyclonal B cell responses (9–11), changes in splenic microarchitecture (12) and B cell compartments such as germinal centres (13), and deletion of malaria-specific B cells after acute infection (14), suggest that the B cell response and resulting memory to *Plasmodium* may be impaired. However, *Plasmodium chabaudi* infection in mice does stimulate significant malaria-specific antibody responses, and we have shown that kinetics of MSP-1 specific antibody production on re-challenge are enhanced, a result compatible with the normal generation of memory B cells (15). Studies of the nature of the plasma cell response will be important to discern reasons for the short-lived components of the antibody response to human malaria (8).

Although the exact role for antigen, or pathogen persistence in the humoral memory response is still a matter of debate (16–20), immunity appears to be enhanced in malaria and other infections by pathogen persistence (21–24), suggesting that B cell responses in malaria could be altered by chronic infection. Persistent, low-level parasitaemia following acute infection for up to 3 months is a feature of *P. chabaudi* infection of mice (15), and in this respect is similar to some human infections. The chronic phase of primary infection has been shown to affect both the magnitude of the challenge infection, and the secondary antibody response (15), and so may well have affects on the nature and magnitude of a secondary B cell response. Thus this model may provide some clues about the effects of persistent parasites on the B cell response, which could be applicable to human infection.

A detailed study of the types of B cells activated and plasma cells generated and maintained in this model may help to define the nature of the B cell response and identify populations of antibody-producing cells that are activated and maintained in infection. Therefore, in this study, we have characterized subsets of B cells and plasma cells in primary and challenge infections with *Plasmodium chabaudi*, and have investigated the effect of persistent infection on these subsets. We show here that previously activated B cells and plasma cells survive for 2 months after infection, even after the chronic infection has been eliminated with chloroquine. After re-challenge, in contrast to the primary infection, there is an enhanced B cell response, a greater proportion of mature plasma cells; and splenomegaly and bone marrow depletion are minimal. These data suggest that there is a more focused and efficient secondary response to the parasite, consistent with a memory response.

## MATERIALS AND METHODS

### Mice and parasites

C57Bl/6 mice were bred in the National Institute for Medical Research under SPF conditions and for experiments maintained conventionally with sterile food and irradiated water *ad libitum*. All experiments conducted under British Home Office regulations. Female 5–8-week-old mice were infected with 10^5^ parasitized erythrocytes from *P. chabaudi chabaudi*(AS) infected mice, and monitored by examination of Giemsa-stained blood films as described previously (25). Chronic infection was eliminated by three i.p. injections of 50 mg chloroquine (Sigma, UK)/kg body weight (average 20 g) in 0·9% saline solution at 2-day intervals, from day 30 of infection. This clone of *P. chabaudi* is sensitive to chloroquine when used at low parasite density ((26) and W. Jarra, personal communication); after treatment no parasites were detectable by thin or thick blood film analysis or after sub-inoculation of blood into naïve recipients (15). Primary and secondary infections were conducted simultaneously with age-matched uninfected controls and oldest uninfected mice are shown as day 0.

### Antibodies and flow cytometric analysis

Spleens or bone marrow cells from femurs and tibias, were collected and dissociated into single cells in HBSS (Gibco, UK) containing 5% FBS (Seralabs, UK) and 6 mm HEPES. Erythrocytes were lysed using hypotonic lysis solution (Sigma). Cells were counted (Scharfe System CASY1, Reutlingen, Germany). Subset numbers were calculated by multiplying the percentage of lymphocytes by total number of viable nucleated cells.

**Figure 1:**
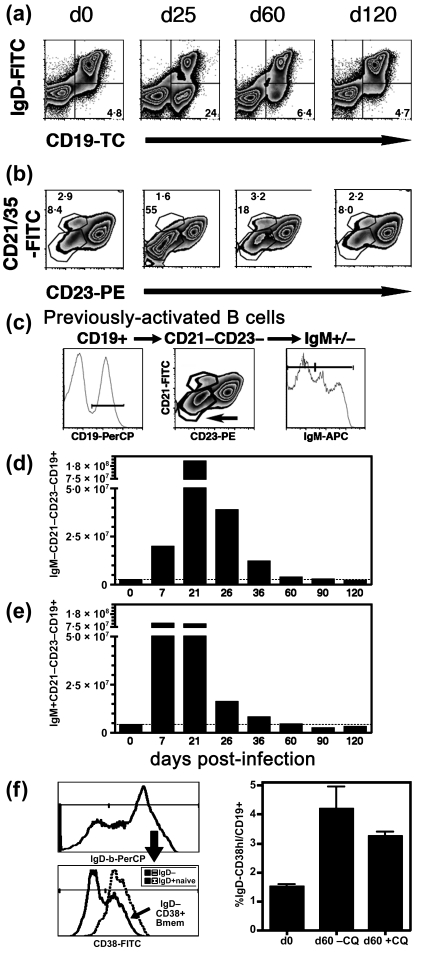
B cells are activated in *P*. *chabaudi* infection and produce memory phenotype cells. C57Bl/6 mice were infected with 10^5^ *P. c. chabaudi*, some were treated with chloroquine (CQ) days 30–34. Splenocytes were analysed by FACS and gated by forward and size scatter properties for the characteristics of lymphocytes. On various days of infection, splenocytes were stained for and gated on CD19-TC and (a) IgD-FITC or (b) CD23-PE, and CD21/35-biotin or (c–e) CD19-TC, CD23-PE, CD21/35-biotin and IgM-APC. (c) Cells were gated as shown for quantitation of memory phenotype cells on CD19+, CD21^−^CD23^−^ and IgM+ or IgM^−^. Day 60 splenocytes are shown. (d) CD19+CD23^−^CD21^−^IgM^−^, and (e) CD19+CD23^−^CD21^−^IgM+ B cells are quantified for four months post-infection. (f) Splenocytes are stained for CD19-TC, IgD-biotin and CD38-FITC and gated on CD19+, as shown in (c, left, day 60). As shown, gates were set according to the levels of CD38 and IgD on naive B cells (left), and memory B cells are quantified as CD38hi IgD-CD19+ (right). Representative plots of five mice shown.

Cells were stained at 3 × 10^6^/well in 96-well V-bottom plates and incubated with anti-CD16/32(24G2) at 37°C for 20 min, followed by 20 min at 4°C to eliminate surface binding of endogenous antibody. After washing, cells were incubated in PBS with 2% FCS and 0·01% Sodium azide and indicated combinations of FITC-, PE-, PerCP, TriColor-, biotin- or allophycocyanin- (APC)-conjugated antibodies with Strepdavidin -FITC or -APC (BD Biosciences, Cambridge Biosciences Oxford, UK). After washing, cells were fixed overnight with 2% paraformaldehyde in PBS. CD138/syndecan-1(281-2), stains were performed in PBS, 1% BSA. For intracellular staining, after surface staining, cells were fixed with PharMingen Cytofix/Cytoperm solution (BD Biosciences). Fixed cells were permeabilized by washing in Perm/Wash buffer (BD Biosciences) twice and 20-min incubation. Cells were stained with goat antimouse-IgG plus IgM-FITC (antiserum, multiply adhered, BD Biosciences) at 70 µg/µL for 3 × 10^6^ cells for 40 min. Cells were washed thrice in Perm/Wash buffer and re-suspended in staining buffer. A total of 30 000 lymphocytes or 1–2 million events were collected for rare cells. Data acquired on a FACS calibur using Cell Quest Pro (Becton Dickenson) and analysed using FlowJo (Portland, OR). For all analyses, cells were first gated by forward and side scatter properties for the characteristics of lymphocytes as shown in Figure 1(c). Plasma cell live gate includes larger cells, but not granulocytes, as some plasma cells are found near the top of the SSC scale. Stains done on different days were synchronized for analysis by equalizing gates in uninfected mice. Plasma cells gated on intracellular IgG/M were drawn after analysing surface staining. Mean fluorescence intensity of surface staining was a log lower than intracellular staining in CD138^hi^CD9^+^B220^−^ plasma cells and thus surface staining cells could be excluded.

### Statistics

All experiments were analysed by one-way ANOVAs and where differences were real, individual groups were studied by Two-tailed, unpaired *t*-tests and *P* ≤ 0·05 considered significant (Prism, GraphPad San Diego, CA).

## RESULTS

### Persistence of previously activated and isotype-switched memory B cells in *Plasmodium chabaudi* infection

The kinetics of parasitaemia and the expansion of splenocytes and B cells in a primary infection were investigated (Supplementary Figure 1). In order to study B cell subpopulations and plasma cells after primary *P. chabaudi*infection, the activation phenotype of B cells was investigated for four months following infection (Figure 1a–e). As B cells are activated, they down-regulate IgD expression and can then differentiate into antibody-secreting cells, or switch isotypes from IgM to IgG in the germinal centre. Down-regulation of IgD on CD19^+^ splenocytes is shown in Figure 1(a). While the percentage of IgD^−^ CD19^+^ B cells increased considerably around day 25, the proportion of previously activated IgD^−^ B cells was only slightly elevated at 2 months and back to baseline by 4 months after infection (Figure 1a). Previously activated B cells have lower levels of expression of the low affinity IgE receptor, CD23 (27,28). We observed that all CD23^−^ B cells during acute infection were also negative for the complement receptor-1/2 (CD21/35). We investigated the generation and maintenance of activated B cells in a time-course of CD19^+^CD21^−^CD23^−^ cells, Figure 1(b). CD21^−^CD23^−^ B cells peak during infection, however, they return to basal levels by 4 months after infection. The percentage of IgM^−^, isotype-switched B cells changed considerably from day 0 to day 60 (from 32% to 59%, IgM^−^, data not shown), so we looked at IgM expression on activated B cells. The gating is shown in Figure 1(c). While IgM^−^ CD21^−^CD23^−^CD19^+^ B cells peak at day 21 and are detectable until day 36 post-infection, IgM^+^ cells peak earlier. This suggests that the IgM^+^ B cells switch isotypes (Figure 1d,e), with IgM^+^ cells increasing first in infection, and IgM^−^ cells appearing dramatically around day 21. Another marker of activation is CD38, which is reduced as IgD is down-regulated on germinal centre B cells and is re-expressed after the germinal centre (GC) reaction on memory B cells (Bmem), but not mature plasma cells in mice (29). In our experiments (Figure 1f), the proportion of IgD^−^CD38^hi^, previously activated but resting memory B cells within the CD19^+^ B cell population, increased from d0 (1·52% CD19 ± 0·07 SEM, *n* = 5) vs. d60 (4·18% ± 0·86, *n* = 5).

**Figure 2:**
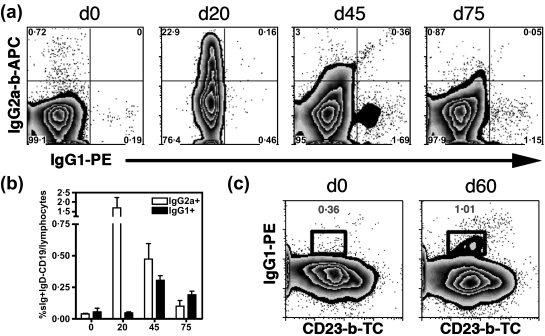
Isotype prevalence changes throughout *P. chabaudi* infection and IgG1 memory cells survive. Mice were infected with *P. chabaudi* and splenocytes analysed by FACS. Density plots are gated on CD19-TC+IgD-FITC- B cells as shown in Figure 1a (LR quadrant). (a) IgG2a-biotin, IgG1-PE staining on various days. (b) Quantification of B cells expressing IgG1 and IgG2a throughout infection. (c) IgG1-PE and CD23-biotin shows that the expanded IgG1^+^ population is CD23^−^. Error bars represent SEM of 3 (d75) -5 mice per group.

To investigate the immunoglobulin isotype on B cells in this infection, we examined surface expression of IgG2a and IgG1 on CD19^+^ IgD^−^ cells. By 20 days post-infection, a dramatic increase in the proportion of IgG2a^b^ + B cells (22·9% of CD19^+^IgD^−^ in Figures 2aand 1·7 ± 0·05% of lymphocytes Figure 2b) was observed, which diminished by day 45, when an IgG1^+^ population was detectable. Approximately 30% of remaining IgD^−^ B cells, two months after infection, expressed IgM; however, isotype of the Ig on the remaining CD19^+^IgD^−^ cells could not be determined. At day 60, a distinct population of IgG1^+^CD23^−^CD19^+^ previously activated or memory B cells was observed (Figure 2c). The appearance of IgG1^+^ cells after IgG2a agrees with earlier observations on serum antibody kinetics in *P. chabaudi* infections, and coincides with the gradual increase in malaria-specific IgG1 antibodies and T helper cell precursors and the gradual decrease in IgG2a serum antibody and IFN-γ producing Th1 cells observed in this infection (30). However, this analysis represents both malaria-specific and bystander activation of B cells.

### B cells respond differently in a second infection

**Figure 3:**
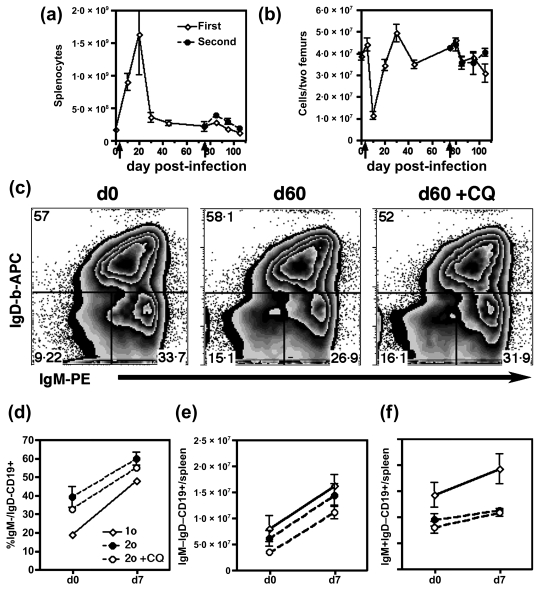
Increase in proportion of isotype-switched B cells after infection and lack of re-expansion of IgM^+^IgD^−^ B cells. Mice were infected and some were treated with chloroquine (CQ) days 30–34 post-infection. Two months post-infection, they were re-infected and analysed 7 days later (2^o^ d7). (a) Splenocytes and (b) Bone marrow cells were counted during the course of a first or second infection (c) Splenocytes were stained and analysed by FACS for CD19-TC, IgD-biotin, and IgM-PE on days 0, 60 and 7 (not shown) of the primary and secondary infection. Plots are gated on CD19, as shown in Figure 1C and (c) Percent of total CD19^+^ cells is shown. (d) Percent of activated B cells, CD19^+^IgD^−^ that are isotype switched, IgM^−^ and (e) numbers of isotype switched, IgM^−^IgD^−^ and (f) non-isotype switched, but previously activated B cells, IgM^+^IgD^−^, per spleen. Error bars show SEM for 4–5 mice/time point. ** *P* ≤ 0·01, *** *P* ≤ 0·001

We next examined the phenotype of B cells after a second infection with *P. chabaudi*, to determine whether activation or expansion of different subpopulations took place more rapidly, as expected for a memory response. Since a primary *P. chabaudi* infection in C57Bl/6 can remain chronic, at very low levels, for up to 60 days, re-infection of mice in this time superimposes a second infection over the existing chronic primary infection. We therefore determined whether there was a rapid expansion of the different B cell populations, a defining characteristic of an adaptive recall response, on second challenge with *P. chabaudi* given to chronically infected mice and to mice treated with chloroquine after the primary infection to eliminate parasites. As described previously, both groups of mice were partially immune to the second infection, with lower secondary parasitaemias in re-challenged, chronically infected mice (15). Although splenomegaly is dramatic in the first infection, there was only a small increase in spleen cellularity following the second infection (Figure 3a). Furthermore, while more than 50% of bone marrow cells are mobilized out of the bone marrow at the peak of the first infection, a second infection does not seem to impact bone marrow cellularity (Figure 3b).

Activated and isotype-switched B cells (CD19^+^IgD^−^IgM^−^, Figure 3c–f), as well as marginal zone and IgM^+^ memory B cells (CD19^+^IgD^−^IgM^+^) were also measured after the second infection (Figure 3c). As an indication of memory B cell re-activation, the speed of the B cell response was compared in the first and second infections. The proportion of isotype-switched B cells (%IgM-/IgD-CD19+) doubled at day 60 suggesting a change in the composition of the B cell pool towards more isotype-switched cells (Figure 3d), however, neither IgM^+^ nor IgM^−^ IgD^−^ B cell numbers were significantly increased at day 60 (Figure 3e,f). Interestingly, looking at expansion from day 0 of primary or secondary (day 60) infection, it is clear that upon re-challenge, the IgM^−^, isotype-switched memory cells, but not the IgM^+^ IgD^−^ previously activated population expanded significantly by day 7 (Figure 3e,f). This may be explained by data showing that expansion of IgM^+^ memory cells is inhibited by signalling of antigen-specific IgG via their Fc Receptors (31), or that these cells switch isotype quickly in the second infection.

### Dramatic reactivation of memory B cells in the germinal centre and marginal zone subsets in second infection

**Figure 4:**
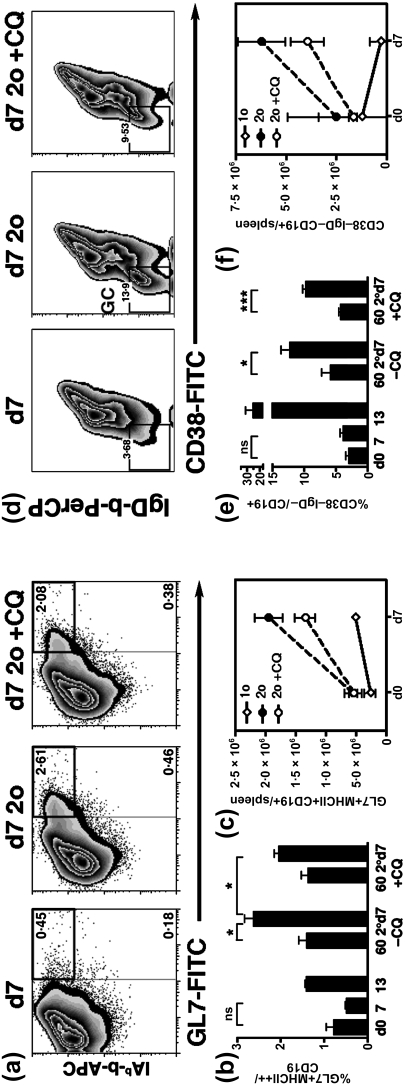
Germinal centre cells expand substantially in the second infection. Mice were infected and some were treated with chloroquine (CQ) days 30–34. Two months later (d60), they were re-infected and analysed (2^o^ d7). Splenocytes were stained and analysed by FACS for (a–c) CD19-TC, GL7-FITC and I-A^b^ (MHCII)-PE or (d–f) CD19-APC, CD38-FITC and IgD-biotin. (a, d) Plots are gated on lymphocytes and CD19^+^ as shown in Figure 1c. (b) Percent GL7^+^MHCII^++^ of CD19^+^ germinal centre B cells. (c) Expansion of Germinal centre cells is seen from the numbers of CD19^+^GL7^+^MHCII^++^ cells d0, d7, d60 (d0 2°), and d7 of the second infection. (e) Proportion and (f) number of germinal centre B cells, CD38^−^ IgD^−^ of CD19. Plots are representative of five mice. Error bars show SEM for 4–5 mice/time point. * *P* ≤ 0·05, ** *P* ≤ 0·01, *** *P* ≤ 0·001, ns = not significantly different, 2°= secondary.

Measurement of germinal centre B cells was included in the analysis of primary and secondary responses, as the germinal centre (GC) is the site of the T-cell-dependent B cell response, which includes expansion of antigen-specific clones, affinity maturation of the immunoglobulin receptor and isotype switching, resulting in the generation of memory B cells and plasma cells. Memory B cell re-activation can be seen here by comparing B cell expansion in the germinal centre in a first infection to that in a second. Activated, GL7^+^MHCII^hi^, germinal centre B cells were measured at day 0 and day 7 of the first and second infection. The effect of drug elimination of the primary infection on the secondary B cell response was also compared (Figure 4). In a primary infection, the numbers and proportions of germinal centre B cells (CD19^+^GL7^+^) increased with kinetics similar to other activated B cell subsets with a maximum of 8·9% (day 25 ± 1·8%) of all CD19^+^ B cells and returned to the level of age-matched, uninfected mice by 90 days (data not shown). While the germinal centre B cell population was not significantly above the levels of uninfected mice on day 7 in the primary infection (Figure 4a,b), expansion of GL7^+^MHCII^hi^ B cells was significantly greater in a second infection, as seen on day 7 (Figure 4a–c), with an increase in both the percentage of GL7^+^ cells and an almost fourfold increase in numbers (Figure 4b,c), suggesting an adaptive memory response. Figure 4d–f show this increase in B cell activation in the challenge infection using the phenotype CD38^−^IgD^−^ to identify germinal centre B cells. CD38 is down-regulated on activation and returns to normal levels on memory B cells (29) and identifies activated B cells slightly earlier than GL7. CD38 down-regulation shows a dramatic difference between the first and second infection in percent (Figure 4d,e) and numbers (Figure 4f) and a dramatically increased rate of expansion can be seen in Germinal Centre B cell population in the second infection (Figure 4c,f). The increase in germinal centre cells in second infection was not due to increased levels of antigen, as on day 7 of a second infection, parasitaemia was one to two orders of magnitude lower than in a primary infection (15). Chloroquine treatment at 30 days post-primary infection did not affect numbers or proportions of GC B cells before re-challenge (d60), but at day 7 after re-infection there was a lower percentage and number of GC B cells (Figure 4b,c, *P* = 0·02, and 0·03, respectively) in the drug-treated mice.

**Figure 5:**
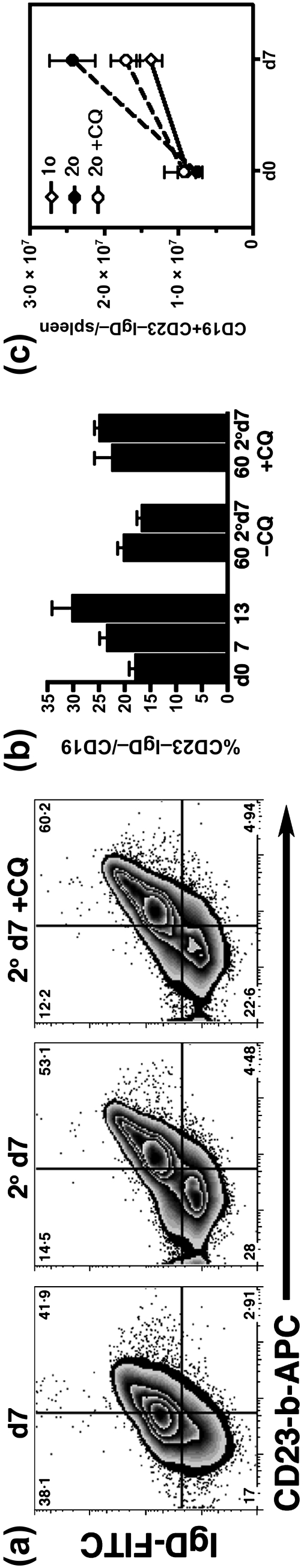
Less CD23 down-regulation in second infection and expansion of Marginal Zone B cells. Mice were infected and some were treated with chloroquine (CQ) days 30–34 post-infection. Two months post-infection, they were re-infected and analysed 7 days later (2^o^ d7). (a) Splenocytes were stained and analysed by FACS for CD19-TC and IgD-FITC, and CD23-biotin on day 7 of the primary and secondary infection. (b) Percent and (c) numbers of CD23-IgD-CD19^+^ cells. Error bars show SEM for 3–5 mice/time point.

As the Marginal Zone also contains memory B cells, it was important to ascertain their participation in the second infection. Marginal Zone B cells can be detected using CD23 and IgD down-regulation. While CD23 is much less down-regulated overall on B cells in second infection (Figure 5a, left quadrants), there is a stronger expansion of CD23^−^IgD^−^ B cells on re-challenge (Figure 5a–c). There was no significant difference in marginal zone B cell numbers, measured as either IgM^+^IgD^−^ or CD23^−^IgD^−^ B cells between drug-treated or chronically infected mice.

### More B220^−^ mature bone marrow plasma cells produced in second infection

**Figure 6:**
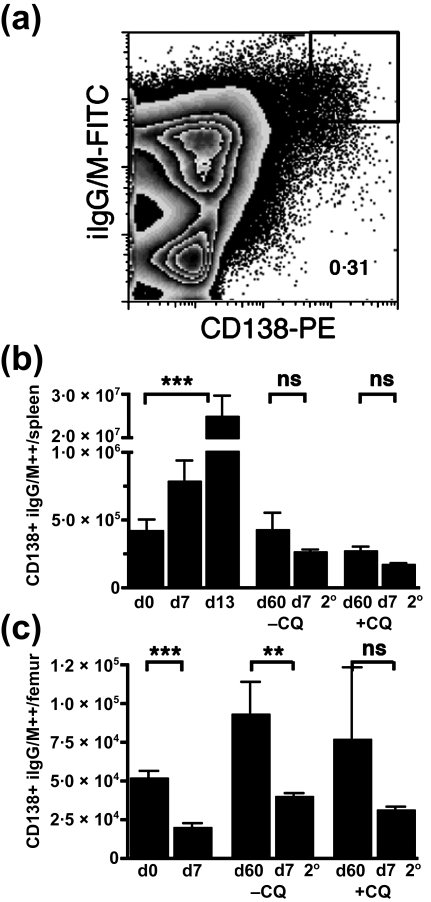
Second infection gives rise to fewer splenic and more bone marrow plasma cells. Mice were infected and some groups were treated with Chloroquine (CQ). Two months post-infection (d60) they were re-infected (2^o^) and analysed for plasma cells. (a, b) splenocytes or (c) BM cells were stained for CD138-PE, intracellular anti-IgG^+^IgM-FITC, B220-APC, CD9-biotin. Density plot gated on live cells, excluding granulocytes. (a) Representative plot of splenocytes day 7 post-infection. Numbers of plasma cells (b) per spleen or (c) two legs. Error bars show SEM for 3 (d60) or 5 mice/time point. ** *P* ≤ 0·01, *** *P* ≤ 0·001, ns = not significantly different.

To determine whether the increase in germinal centre and marginal zone activity corresponded with an increase in mature plasma cell production on second infection, we examined plasma cells in the spleen and bone marrow (BM) of infected mice. In agreement with our previous findings (12), there was a large transient increase in splenic plasma cells, which expressed high levels of Syndecan-1 (CD138) as well as intracellular IgG or IgM (iIgG/M) (32) (gated as shown in Figure 6a), in the acute phase of primary infection (Figure 6b), peaking during acute infection and returning to levels of uninfected controls by day 60. The antibody used to detect intracellular antibody in plasma cells recognizes both IgG and IgM, however, we do not suggest that these isotypes can be expressed simultaneously. However, at day 7 of re-infection, plasma cell expansion was not observed in the spleen (Figure 6b). Plasma cell numbers in the bone marrow show a twofold loss of CD138^hi^ plasma cells at the peak of infection (Figure 6c), corresponding with overall loss of bone marrow cells. But strikingly, more bone marrow plasma cells are apparent at day 7 of the second infection than in the first. Chloroquine treatment had no significant effect on total number of plasma cells in spleen or BM at day 60 of primary infection.

In order to characterize the plasma cell subpopulations in the spleen and BM in *P. chabaudi*, we examined the expression of CD9 and B220 on CD138^hi^, intracellular IgG/M^hi^ plasma cells. Syndecan-1^+^ plasma cells can be either short-lived antibody producing cells (B220^+^), long-lived mature plasma cells (B220^−^), or their precursors (B220^+^) (33–35). CD9 is present on plasma cells from both T-dependent and thymus independent (TI-2) responses in C57Bl/6 mice (36).

**Figure 7:**
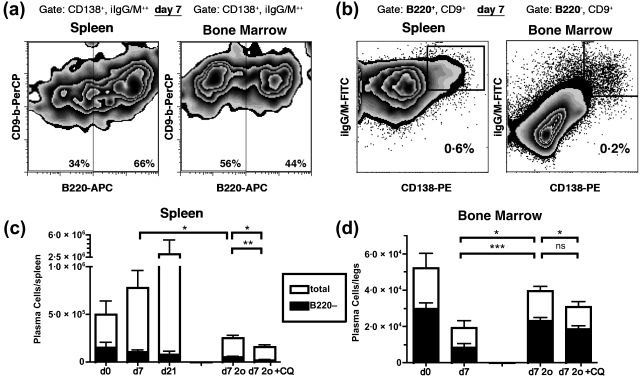
Second infection generates mature bone marrow plasma cells. Mice were infected and some treated with Chloroquine (CQ). Two months post-infection (d60) they were re-infected and analysed for plasma cells (2^o^ d7). (a–d) Spleens and BM cells were stained for CD138-PE, intracellular anti-IgG^+^IgM-FITC, B220-APC, CD9-biotin. (a) Plots gated on live cells, excluding granulocytes, *and all plasma cells*, which are intracellular IgG^+^M^hi^ and CD138hi, or (b) first gated on B220^+^CD9^+^ for splenocytes or B220^−^CD9^+^ for bone marrow, for a plot of the predominant plasma cell type. Numbers on plots represent percent of live cells in region (c, d) Total plasma cell numbers and B220^−^ mature plasma cells are shown. Plots are representative of 5 mice at all time points, and error bars represent SEM. * *P* ≤ 0·05, ** *P* ≤ 0·01, *** *P* ≤ 0·001, ns = not significantly different.

Plasma cells in the spleen and BM, defined as CD138^hi^, iIgM/IgG^hi^, and gated as shown in Figure 6(a), expressed CD9; however, the proportion expressing B220 clearly differed in the two organs and throughout infection. The majority of the plasma cells in the spleen expressed B220 (68% average, day 7 Figure a,b; left panel, B220^+^CD9^+^ gated, 0·6% of splenocytes). In the BM, B220^−^ plasma cells were the majority (Figure 7a,band 0·2% bone marrow cells). In the first infection, most plasma cells were produced in the spleen, and only B220^+^ plasma cells increased in numbers (Figure 7c). Naïve mice also have a majority of B220+ plasma cells in the spleen and B220- in the bone marrow, and percentages of total plasma cells in the bone marrow do not change dramatically, due to tight constraints on the availability of niches (34). By contrast, in the second infection there was no expansion of plasma cells in the spleen, but rather a decrease in numbers compared with uninfected controls (*P* = 0·01). In the bone marrow, reduction of plasma cells was much less marked upon re-challenge than in the primary (Figure 7d). There were more mature B220^−^ (*P* = 0·0002) and total plasma cells (*P* = 0·03) present at day 7 of the second infection compared with the primary infection day 7 (Figure 7d), suggesting that the primary infection generates memory B cells that produce plasma cells on re-infection. Chloroquine treatment resulted in a small but significant reduction in the number of total plasma cells (*P* = 0·02 *t*-test) as well as B220^−^ plasma cells in spleen on reactivation (*P* = 0·002 *t*-test), and on total (*P* = 0·03 *t*-test) but not B220^−^ plasma cells in the BM (Figure 7d), confirming that the B220^−^ subset is not affected by antigen persistence.

## DISCUSSION

Here we have shown that B cells with memory cell characteristics are generated after a single infection of the malaria parasite, *P. chabaudi*. These CD19^+^ cells do not express IgD, CD21 or CD23, though they do express CD38 indicating that they are resting memory cells (29). These surviving cells are either IgM^+^ or isotype switched, some expressing IgG2a^+^ or IgG1^+^. Various subsets of B cells expand rapidly on re-infection. Germinal centre B cells expand dramatically in the first week after re-infection and isotype-switched B cells expand, although cells with the phenotype of IgM^+^ memory cells do not. Marginal Zone B cells also expand, as would be expected from a population reported to contain memory B cells. In addition, despite the large increase in immature B220^+^ plasma cells in the spleen in the primary infection, the second infection saw fewer of these produced. There were almost no immature, B220^+^, plasma cells generated in the second infection, but an increase in bone marrow plasma cells suggests an improved response. These observations are in line with our previous work that demonstrated both faster and greater serum IgG responses to specific parasite antigens following re-infection and that serum levels of specific antibody are maintained for 8 months post-infection (15). The plasma cells and memory-phenotype cells measured here are probably the cells responsible for the anamnestic responses measured in these antigen-specific assays.

A recent report has shown the presence of memory-phenotype B cells to day 40 post-infection (11). Our results support and extend this observation by showing that they survive day 60 post-infection. Looking at a second infection to test the functionality of memory B cells on re-stimulation, we have shown that the B cell response in the second infection has increased kinetics of activation and identified the marginal zone and the germinal centre B cells as the location for memory B cell activation. We have also shown in previous work that specific antibody production is improved in second infection (15), as would be expected from functional memory B cells. Furthermore, we have shown important differences in bone marrow plasma cell production between the primary and secondary responses, which make the memory response more efficient.

In this study we have observed both IgM^−^ and IgM^+^CD19^+^ memory B cells in the spleen, which do not express IgD, CD21 or CD23, and do express CD38. A proportion of the IgM^−^ cells may be isotype-switched memory B cells, and in line with this, the IgM^−^ B cells expanded on re-infection. Interestingly, an IgM^+^ subset of B cells with phenotypic markers of memory cells (IgD^−^CD21^−^CD23^−^) was also persistent, suggesting that in this infection there are IgM^+^ memory B cells (37). The phenotype resembles IgM^+^ memory cells reported in human (37) or marginal zone cells. However, on re-infection, these IgM + B cells either switched isotype, becoming unrecognizable, or did not expand after a second infection. The IgM^+^ memory cells we see here also resemble T-I memory B cells (CD19^+^CD23^−^CD21^−^IgM^+^), which unlike their IgG^+^ counterparts, do not expand on re-challenge in the presence of antigen-specific IgG (31), as we see here (Figure 4c). Both the IgM^+^ and IgM^−^ memory subsets do not express CD21/35. This down-regulation of CR1/2 has also been described in *P. falciparum* infection (in humans, complement receptor 1). It is of interest then that haematin, a malaria parasite product, induces C3 degradation and deposition, and that its concentration correlates with surface expression of CR1 on B cells (38). CD21 has also been reported to be down-regulated after binding to complement-coated pathogen in HIV infections (39).

Our data shows a short-lived expansion of B220 + splenic plasma cells followed by re-population of the BM by a majority of mature B220^−^ plasma cells, suggesting that a fraction of the response is short-lived (35). The bone marrow, during the acute phase of a primary infection, in contrast to the spleen, has reduced numbers of total cells and plasma cells. It has previously been reported that *P. yoelli* infection results in loss of bone marrow plasma cells generated by vaccination (14). This may be as a result of apoptosis *in situ,* or mobilization of resident plasma cells to sites of inflammation such as the spleen (40). In the second infection there was significantly less splenomegaly, less B cell expansion and fewer plasma cells in the spleen than during the acute primary infection. Similarly, bone marrow cellularity is not as reduced as it is in the primary response and more terminally differentiated B220^−^ plasma cells remained, suggesting that the large B cell response in a primary infection contained memory B cells that expanded on re-infection to produce plasma cells. It is likely that it is these cells, together with low-level re-stimulation and differentiation of memory B cells that maintain the serum antibody levels seen up to eight months in this infection (15). Direct studies of plasma cell longevity are currently underway. This could be critical for immunity to re-infection, as BM plasma cells, having undergone significant selection, are more likely to produce the high affinity, neutralizing antibodies important for clearance of the parasite.

Throughout this study, small but significantly greater B cell responses were observed in the second infection of chronically infected mice, with more GL7 + (GC) B cells, and more BM and splenic plasma cells generated compared with drug-treated mice (Figures 4 and 7). These differences cannot be attributed directly to parasite numbers in the second infection, as they are lower in chronically and re-infected mice (15). Therefore, our data suggest that at some level, the presence of persistent parasites, and/or the prolonged presence of antigens from dead parasites, enhances the memory B cell compartment.

In conclusion, we have shown that the B cell response to a second infection is more efficient than the primary response, the definition of adaptive immune memory. Germinal centre B cells, marginal zone, and BM plasma cell populations increase with enhanced kinetics. The faster secondary B cell response, together with reduced splenomegaly and less overall B cell activation, suggests that the B cell response in the second infection is more focused or efficient. The next steps will be to determine whether enhanced responsiveness and changes in B cell and plasma cell phenotypes in spleen and bone marrow reflect the malaria-antigen specific response.

## Supporting Information

**Figure S1** Time-course of parasitaemia and cell numbers. These drawings show the course of parasitaemia and expansion of splenocytes and B cells in the spleen, as measured by flow cytometry of CD19 + cells. They represent data summarized from Achtman *et al.* (2007).
