# Supplementary figures and images for: Germinal centre and marginal zone B cells expand quickly in a second Plasmodium chabaudi malaria infection producing mature plasma cells
Source: Parasite Immunol. 2009 Jan;31(1):20–31. doi: 10.1111/j.1365-3024.2008.01066.x (PMC2680269; doi:10.1111/j.1365-3024.2008.01066.x)

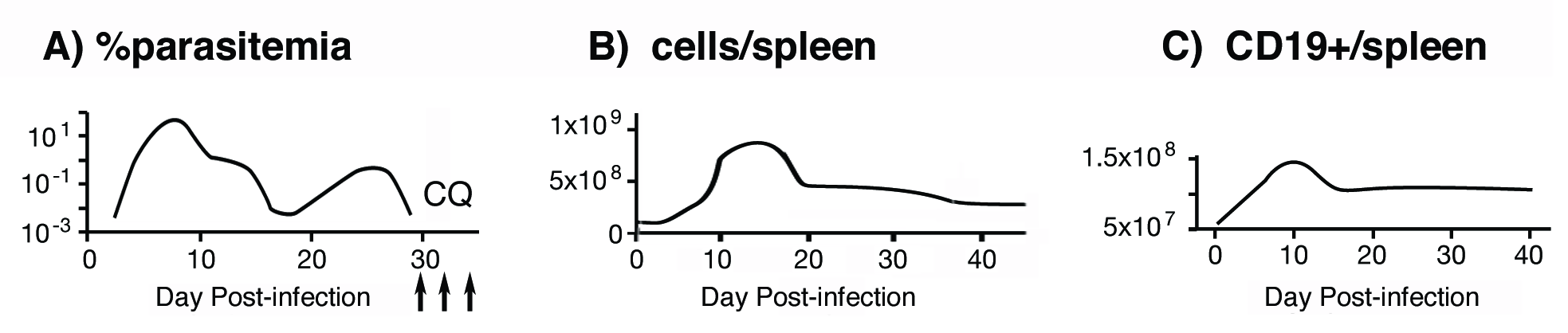

Supplement: Supplementary file 1 [file pim0031-0020-SD1.tif]
